# A Predictive Nomogram of Early Recurrence for Patients with AFP-Negative Hepatocellular Carcinoma Underwent Curative Resection

**DOI:** 10.3390/diagnostics12051073

**Published:** 2022-04-25

**Authors:** Wencui Li, Lizhu Han, Bohan Xiao, Xubin Li, Zhaoxiang Ye

**Affiliations:** 1 Department of Radiology, Liver Cancer Center, Tianjin Medical University Cancer Institute and Hospital, Huanhu Xi Road, Tiyuan Bei, Hexi District, Tianjin 300060, China; 15655332015@163.com (W.L.); hanlizhu2015@163.com (L.H.); xiaobohantj@163.com (B.X.); 2 Key Laboratory of Cancer Prevention and Therapy, National Clinical Research Center for Cancer, Tianjin 300060, China; 3 Tianjin’s Clinical Research Center for Cancer, Tianjin 300060, China

**Keywords:** hepatocellular carcinoma, alpha-fetoprotein-negative, early recurrence, nomogram

## Abstract

Background: Alpha-fetoprotein-negative (<20 ng/mL) hepatocellular carcinoma (AFP-NHCC) cannot be easily diagnosed in clinical practice, which may affect early treatment and prognosis. Furthermore, there are no reliable tools for the prediction of AFP-NHCC early recurrence that have been developed currently. The objective of this study was to identify the independent risk factors for AFP-NHCC and construct an individual prediction nomogram of early recurrence of these patients who underwent curative resection. Methods: A retrospective study of 199 patients with AFP-NHCC who had undergone curative resection and another 231 patients with AFP-positive HCC were included in case-controlled analyses. All AFP-NHCC patients were randomly divided into training and validation datasets at a ratio of 7:3. The univariate and multivariate Cox proportional hazards regression analyses were applied to identify the risk factors, based on which the predictive nomogram of early recurrence was constructed in the training dataset. The area under the curve (AUC), calibration curve, and decision curve was used to evaluate the predictive performance and discriminative ability of the nomogram, and the results were validated in the validation dataset. Results: Compared to AFP-positive patients, the AFP-negative group with lower values of laboratory parameters, lower tumor aggressiveness, and less malignant magnetic resonance (MR) imaging features. AST (HR = 2.200, *p* = 0.009), tumor capsule (HR = 0.392, *p* = 0.017), rim enhancement (HR = 2.825, *p* = 0.002) and TTPVI (HR = 5.511, *p* < 0.001) were independent predictors for early recurrence of AFP-NHCC patients. The nomogram integrated these independent predictors and achieved better predictive performance with AUCs of 0.89 and 0.85 in the training and validation datasets, respectively. The calibration curve and decision curve analysis both demonstrated better predictive efficacy and discriminative ability of the nomogram. Conclusions: The nomogram based on the multivariable Cox proportional hazards regression analysis presented accurate individual prediction for early recurrence of AFP-NHCC patients after surgery. This nomogram could assist physicians in personalized treatment decision-making for patients with AFP-NHCC.

## 1. Introduction

Hepatocellular carcinoma (HCC) is one of the most common histological types of liver cancer, accounting for 90% of primary liver cancers with high morbidity and mortality [1,2]. Surgical resection, liver transplantation, and some locoregional treatment are the main curative treatments for HCC patients in the early stages [3]. Notably, immune checkpoint inhibitors (ICIs) have changed the treatment scenario of unresectable HCC in the last five years [4]. Nonetheless, the prognosis of HCC patients still remains grim due to inefficient diagnosis, especially in early or small HCC and higher rates of early recurrence [5,6].

Alpha-fetoprotein (AFP) is considered a reliable tumor biomarker that is widely used for screening, diagnosing, and monitoring tumor recurrence and metastasis of HCC in daily clinical practice [7,8]. Serum AFP concentration >400 ng/mL is the indication of HCC. A higher AFP concentration is closely correlated with a poorer prognosis, higher aggressiveness of the tumor, and lower response to therapies [9,10]. However, around one-third of HCC patients are defined as AFP-negative HCC (AFP-NHCC) with serum AFP levels < 20 ng/mL, which could affect early diagnosis and treatment [11,12]. Bai et al. [13] reported that patients with AFP-NHCC often had smaller tumor sizes, higher tumor differentiations, and better clinical outcomes. Thus, it is crucial to identify independent risk factors of AFP-negative HCC for the diagnostic and prognostic evaluation in this subgroup.

Several HCC staging systems including the Barcelona Clinic Liver Cancer (BCLC) staging system, Tumor-Node-Metastasis (TNM) staging system, and the Japan Society of Hepatology (JSH) staging system are widely accepted in prognostic evaluation, reasonable treatment option selection, and clinical researches [14]. However, there are varying degrees of defects with these systems in predicting prognosis and providing quantitative risk measures. Some studies have shown that the BCLC score is only suitable for the advanced stages of HCC. As TNM only pays close attention to tumor characteristics rather than liver function, its influence on prognosis remains controversial [15,16]. Therefore, these staging systems are not adequate in predicting early recurrence for AFP-NHCC patients. Novel methods for predicting prognosis and acquiring more precise prognostic information are urgently needed. Recently, the prediction of survival and recurrence of different types of cancers, for instance, urothelial carcinoma, lung cancer, and breast cancer, is convenient with the widespread use of nomograms [17,18,19]. The objective of our study was to identify the independent risk factors among the clinic-pathological factors and MR imaging features, then construct a nomogram for individual prediction of early recurrence in AFP-NHCC patients who underwent curative resection.

## 2. Materials and Methods

### 2.1. Patients

This retrospective study was approved by the ethics committee of Tianjin Medical University Cancer Institute and Hospital, and the written informed consent of each patient was waived. All HCC patients with preoperative AFP who underwent surgical resection at our institution between January 2015 and December 2018 were retrospectively analyzed. The inclusion criteria of patients were: (a) surgical pathology confirmed HCC with margin-negative; (b) clinicopathologic and follow-up information were complete. (c) the surgical resection was performed within 1 month after MRI; (d) the MR images with good image quality were available. The exclusion criteria included: (1) receipt of preoperative anti-HCC treatment; (2) distant metastasis or other malignant diseases. Overall, 430 patients (358 males and 72 females; median age, 59.00 (51.00–64.00) years) met the inclusion criteria and were included in this study. This study included 199 patients with AFP-negative HCC (AFP-NHCC) and 231 patients with AFP-positive HCC. These AFP-NHCC patients were divided randomly into a training dataset (n = 139; 121 males and 18 females; median age: 59.647 (52.384–68.910) years) and a validation dataset (n = 60; 52 males and 8 females; median age 58.617 (49.76–67.474) years) at a ratio 7:3.

### 2.2. Follow-Up

All patients were regularly followed up after discharge. Serum AFP levels, liver function tests, and various imaging examinations (ultrasound, contrast-enhanced CT, or MRI) were performed 1 month after surgery to monitor tumor recurrence, and every 3 or 6 months thereafter. Early recurrence was defined as intrahepatic and/or extrahepatic recurrence of HCC within 2 years after surgery. Recurrence-free survival (RFS) was defined as the interval between the date of surgery and the date of tumor recurrence. 

### 2.3. Clinicopathologic Characteristics

Clinicopathologic characteristics were collected from electronic medical records (Table 1), including age, gender, underlying liver disease, Child-Pugh class, total bilirubin (TBIL), direct bilirubin (DBIL), albumin (ALB), aspartate transaminase (AST), alanine aminotransferase (ALT), γ-glutamyl transferase (GGT), carcinoembryonic antigen (CEA), platelet count (PLT), histologic differentiation, and the status of microvascular invasion, etc. 

### 2.4. MRI Analysis

All MR images were interpreted by 2 radiologists with 5 and 8 years of abdominal MRI experiences, respectively. Both radiologists were aware of HCC but blinded to other information. Discrepancies were resolved through discussion until consensus was reached. The radiologists assessed the following MRI features for each patient: (a) multifocality (0, solitary; 1, multiple); (b) maximum tumor length (0, L-max ≤ 5 cm; 1, L-max > 5 cm); (c) tumor margin (0, smooth margin; 1, non-smooth margin); (d) tumor capsule (0, well-defined tumor capsule; 1, ill-defined tumor capsule); (e) peritumoral enhancement (0, absent; 1, present); (f) rim enhancement (0, absent; 1, present); (g) intratumor necrosis (0, absent; 1, present); (h) intratumor hemorrhage (0, absent; 1, present); (i) Two-trait predictor of venous invasion, TTPVI, (0, TTPVI-absent; 1, TTPVI-present). These MRI features of the largest tumor were recorded when the lesions were multifocal.

### 2.5. Statistical Analysis

Statistical analysis was performed in this study with SPSS (version 26.0, Chicago, IL, USA) and R software (version 4.1.2 (November 2021); http://www.Rproject.org). The *t*-test and Mann-Whitney U test were performed to compare continuous variables between two independent groups; the Chi-square test was used in categorical variables. The selection of independent prognostic factors with the univariate and multivariate Cox proportional hazards regression analysis. The “survival” package, “rms” package, and the “survivalROC” package were used to construct a multivariate Cox proportional hazards model and plot nomogram, calibration curve, and ROC curve. The decision curve was drawn with a “ggDCA” package. A two-tailed *p* < 0.05 was considered to be statistically significant.

## 3. Results

### 3.1. Patient Characteristics

**Figure 1:**
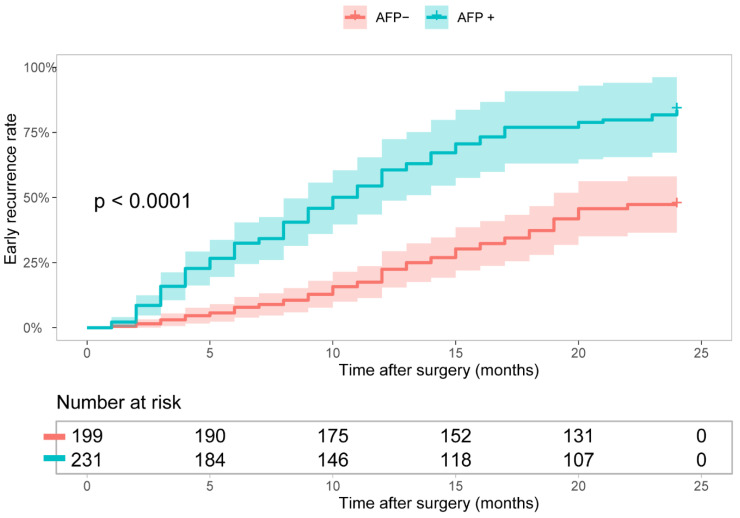
Early recurrence rate of surgically-resected hepatocellular carcinoma (HCC) patients with AFP− vs. AFP+.

The baseline clinicopathological characteristics and MR imaging features of all patients in the study were shown in Table 1. Compared to the AFP-positive group, the patients in the AFP-negative group were older and had lower values of laboratory parameters, lower tumor aggressiveness, and less malignant MR imaging features. These differences in patient characteristics between the two groups may affect overall survival. RFS was longer for AFP-negative patients compared with AFP-positive patients, the early recurrence rates were 37.68% versus 56.70%, respectively (*p* < 0.0001, Figure 1). 

### 3.2. Independent Prognostic Factors of AFP-NHCC Patients

A total of 199 HCC patients with AFP-negative were included in this study, these patients were divided randomly into a training dataset (n = 139, including 50 patients with early recurrence) and a validation dataset (n = 60, including 25 patients with early recurrence) at a ratio 7:3 (Table 2). In the training dataset, univariate Cox proportional hazards regression analysis revealed that AST, CA199, multifocality, tumor margin, tumor capsule, peritumoral enhancement, rim enhancement, and TTPVI were statistically significant predictors of early recurrence (Table 3). All these significant predictors were included in multivariate Cox proportional hazards regression analysis, and the results showed that AST (HR = 1.975, 95%CI: 1.045–3.732, *p* = 0.036), tumor capsule (HR = 0.422, 95%CI: 0.198–0.900, *p* = 0.026), rim enhancement (HR = 2.819, 95%CI: 1.267–6.273, *p* = 0.011), and TTPVI (HR = 11.665, 5%CI: 3.978–34.203, *p* < 0.001) were independent predictors of early recurrence for AFP-NHCC patients (Table 3).

### 3.3. Construction and Evaluation of Nomogram

**Figure 2:**
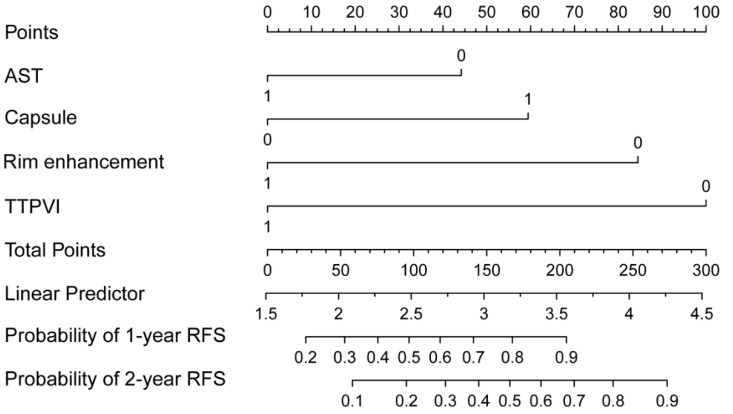
The recurrence-free survival (RFS) nomogram for HCC patients with AFP negative. The nomogram was scaled by the proportional regression coefficient of each predictor. (AST, Aspartate transaminase; Rim enhancement; Capsule, Tumor capsule; TTPVI, two-trait predictor of venous invasion.

**Figure 3:**
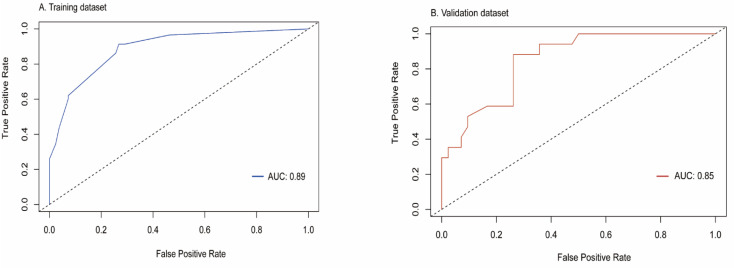
Receiver operating characteristics curve (ROC) for predicting early recurrence of AFP-NHCC patients in the training and validation datasets.

**Figure 4:**
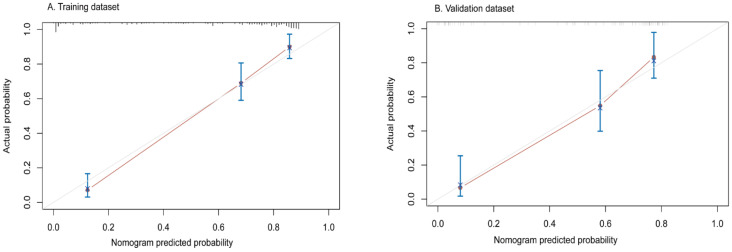
Calibration curve in the training (**A**) and validation (**B**) datasets. The *x*-axis typifies the predicted probability of nomogram for early recurrence, *y*-axis shows the actual early recurrence probability in the patients.

**Figure 5:**
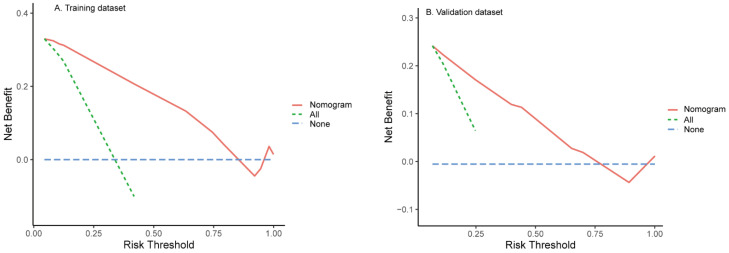
Decision curve for early recurrence in the training (**A**) and validation datasets (**B**). The *y*-axis is the net benefit; the *x*-axis measures the threshold probability. All patients with early recurrence (Green dotted line). No patients with early recurrence (Blue dotted line). The expected net benefit of each patient based on the nomogram (Red line).

All independent predictors were integrated to develop the nomogram in the training dataset (Figure 2). The area under the curve (AUC) of the receiver operator characteristic (ROC) curve and calibration curve was used to evaluate the performance of the nomogram. The AUCs were 0.89 (95%CI: 0.83–0.94) and 0.85 (95%CI: 0.75–0.94) in the training and validation datasets, respectively (Figure 3). The calibration curve indicated that the predicted probabilities of the nomogram were similar to actual early recurrence probabilities in the datasets (Figure 4). The decision curve showed a higher net benefit for early recurrence in the reasonable threshold probability in the training and validation datasets (Figure 5).

## 4. Discussion

In this study, we included a cohort of patients with AFP-NHCC who underwent surgical resection in order to find out the independent risk factors of early recurrence. The results showed that a predictive model derived from AST, tumor capsule, rim enhancement, and TTPVI could be a helpful tool to estimate the probability of early recurrence in this subgroup. It might identify patients with a high risk of early recurrence using our predictive model, for whom liver transplantation, a wider extension of resection, and close follow-up should be considered [20,21]. The nomogram based on the multivariable Cox regression analysis displayed a better predictive performance, with AUC s of 0.89 and 0.85 in the training and validation datasets, respectively. The nomogram demonstrated a higher predicted precision and a better net benefit of early recurrence which were evaluated by the calibration curve and decision curve. The nomogram is a user-friendly graphical tool that can help physicians rapidly compute the probability of early recurrence and make personalized treatment options.

Our nomogram integrated four independent predictive factors for early recurrence, including rim enhancement, tumor capsule, TTPVI, and AST. Some studies have shown that rim enhancement is an important factor for poor prognosis in HCC patients [22,23]. The rim enhancement could be explained by the pathologic features which show the stromal fibrosis in the center of the lesion while the abundant tumoral cellularity in the periphery of the lesion [24]. The rim enhancement indicates infiltrative growth, poor differentiation, and worse prognosis, which may reflect the absence of a tumor capsule and microvascular invasion [25,26]. A tumor capsule is a layer of fibrous structure that limits the aggressiveness and spread of the tumor. Ng et al. reported that encapsulated tumors had a lower incidence of tumor microsatellites and direct liver invasion compared to non-encapsulated ones. Patients with encapsulated tumors tend to have a better prognosis [27]. A tumor capsule is a significant predictive factor of early recurrence in our study, which is in good agreement with the results of a previous study [28]. Segal et al. [29] first discovered that the TTPVI imaging feature might be used for predicting microvascular invasion of HCC. The study showed that TTPVI was associated with a specific HCC molecular profile, which was derived from a venous invasion gene profile related to angiogenesis, cellular proliferation, and matrix invasion. Renzulli et al. [30] confirmed that TTPVI had the same diagnostic accuracy in predicting microvascular invasion on CT and MR imaging. TTPVI can serve as a prognostic marker for HCC after hepatectomy [31]. In our study, TTPVI is also an important risk factor for early recurrence. The AST level is an indicator to activate inflammatory activity which reflects the etiopathogenetic mechanism of hepatocyte necrosis in patients with liver cirrhosis. Cirrhosis and chronic active hepatitis are risk factors for intrahepatic recurrence [32]. Therefore, AST is a significant predictive factor that reflects hepatic inflammation and affects long-term survival [33,34]. The AST level is remarkably associated with early recurrence in multivariate analysis in our study. All HCC patients in our cohort underwent hepatectomy with margin-negative resection confirmed by surgical pathology. Although initial surgical treatment (major or minor hepatectomy) was not an independent risk of early recurrence for AFP-NHCC patients in multivariate Cox analysis. We still think in theory that initial surgical treatment could have an effect on tumor recurrence and overall survival because it was a significant predictor in univariate analysis. It is still controversial for age to be a prognostic factor for HCC patients after hepatectomy [35,36]. In our research, age was significantly different between AFP-negative and AFP-positive groups, the patients in the AFP-negative group were older compared to the AFP-positive group. But the difference in age between recurrence and non-recurrence groups was small in this AFP-negative subgroup. In a word, the characteristics including rim enhancement, tumor capsule, TTPVI, and AST in AFP-NHCC patients should receive adequate attention. The nomogram including the above four predictive factors demonstrated superior discrimination ability of early recurrence in patients with AFP-NHCC. In addition, liver transplantation as an alternative treatment and a shorter interval time of follow-up should be considered for patients with a high risk of early recurrence predicted by the nomogram.

There were several limitations in our study although the nomogram had a better predictive performance. First, it was a retrospective study performed at a single institution, therefore, the selection bias of the predictive nomogram was unavoidable. The prospective studies are needed to further validate this result. Second, the number of patients with AFP-NHCC is limited, and the follow-up time is shorter in our study. Therefore, a larger number of AFP-NHCC patients with five-year follow-up and overall survival time data are required in future studies to verify our results. Third, all patients with AFP-NHCC underwent surgical resection in our study, whether the predictive nomogram would be suitable for patients who received other anti-tumor treatments remains uncertain. Fourth, since the follow-up time is short, it is impossible to evaluate the efficacy of different treatments in patients with tumor recurrence, and it is unknown whether these patients with hepatectomy had a higher overall survival than those who received other treatments, palliative or supportive care.

In conclusion, we used a novel method to construct and validate a nomogram based on the multivariable Cox proportional hazards regression analysis to predict early recurrence in patients with AFP-NHCC after curative resection. The nomogram including rim enhancement, tumor capsule, TTPVI, and AST independent predictive factors displayed a better predictive ability which could assist physicians in personalized treatment decision-making for patients with AFP-NHCC.

## Figures and Tables

**Table 1:** Baseline characteristics of alpha-fetoprotein (AFP)− and AFP+ patients.

Characteristic	AFP− (<20 ug/mL) (n = 199)	AFP+ (≥20 ug/mL) (n = 231)	*p*-Value
Patient demographic			
Age	60.00 (54.00–66.00)	56.00 (49.00–62.00)	<0.001
Gender			0.058
Male	173	185	
Female	26	46	
Liver disease			0.262
HBV/HCV	125	157	
Other	74	74	
Liver cirrhosis			0.018
Present	69	56	
Absent	130	175	
Ascites			0.309
Present	25	37	
Absent	174	194	
Laboratory parameters			
ALT (IU/L)	32.00 (21.00–45.00)	34.00 (24.00–53.00)	0.114
AST (IU/L)	33.00 (25.00–48.00)	38.00 (27.00–55.00)	0.027
ALB	42.50 (40.10–45.20)	42.20 (39.30–44.80)	0.227
TBIL	16.40 (11.90–20.80)	16.30 (13.20–20.80)	0.459
DBIL	3.20 (2.30–4.10)	3.30 (2.50–4.20)	0.239
CR	65.00 (58.00–73.00)	65.0 (57.00–75.00)	0.708
ALP (IU/L)	98.00 (78.00–127.25)	109.00 (89.00–133.00)	0.002
GGT (IU/L)	49.00 (32.00–96.00)	66.00 (39.00–120.00)	0.002
PLT	165.00 (126.00–214.00)	168.00 (122.00–217.00)	0.966
CA199	16.86 (8.70–29.34)	20.91 (11.62–36.32)	0.005
CEA	2.57 (1.58–3.74)	2.67 (1.78–3.79)	0.995
Child-Pugh grade			0.059
A	196	221	
B	3	11	
MRI features			
Multifocality			0.100
Solitary	166	178	
Multiple	33	53	
L-max			0.007
≤5 cm	118	110	
>5 cm	81	121	
Tumor margin			0.006
Smooth	45	29	
Non-smooth	154	202	
Tumor-capsule			0.183
Present	179	198	
Absent	20	33	
Peritumoral enhancement			<0.001
Present	18	50	
Absent	181	181	
Rim enhancement			<0.001
Present	35	79	
Absent	164	152	
TTPVI			0.001
Present	99	151	
Absent	100	80	
Intra-hemorrhage			0.152
Present	33	51	
Absent	166	180	
Intra-necrosis			0.401
Present	63	82	
Absent	136	149	
Histologic characteristics			
Histologic grade			<0.001
Poor	42	92	
Mediate	149	137	
Well	8	2	
Satellite nodules			0.002
Present	27	59	
Absent	172	172	
MVI			<0.001
Present	79	131	
Absent	120	100	
Early recurrence			<0.001
Present	75	131	
Absent	124	100	

ALT = Alanine aminotransferase, AST = Aspartate aminotransferase, ALB = Serum albumin, TBIL = Total bilirubin, DBIL = Direct bilirubin, CR = Creatinine, ALP = Alkaline phosphatase, GGT = γ-glutamyl transpeptadase, PLT = Platelet count, CA199 = Carbohydrate antigen199, CEA = Carcinoembryonic antigen, L-max = Maximum tumor length, TTPVI = Two-trait predictor of venous invasion, MVI = Microvascular invasion.

**Table 2:** Baseline characteristics in the training and validation datasets.

Characteristic	Training Dataset	Validation Dataset	*p*-Value
	n = 139	n = 60	
Patient demographics			
Age (y)	59.647 (9.263)	58.617 (8.857)	0.466
Gender			1.000
Female	18	8	
Male	121	52	
Liver disease			1.000
Hepatitis B/C virus	87	38	
Absent	52	22	
Liver cirrhosis			0.821
Present	47	22	
Absent	92	38	
Ascites			0.654
Present	16	9	
Absent	123	51	
Surgical treatment			
Major hepatectomy	33	20	0.160
Minor hepatectomy	106	40	
Laboratory factors			
ALB (g/L) > 40, ≤40	103, 36	49, 11	0.331
ALT (IU/L) > 50, ≤50	30, 109	14, 46	0.930
AST (IU/L) > 40, ≤40	49, 90	18, 42	0.578
TBIL (μmol/L) > 19, ≤19	50, 89	16, 44	0.265
DBIL (μmol/L) > 3.4, ≤3.4	64, 75	25, 35	0.678
GGT (IU/L) > 40, ≤40	52, 87	32, 28	0.287
ALP (IU/L) > 125, ≤125	38, 101	13, 47	0.507
CR (μmol/L) > 110, ≤110	2, 137	0, 60	0.873
CEA (ng/mL) > 3.4, ≤3.4	42, 97	14, 46	0.412
CA199(U/mL) > 37, ≤37	22, 117	8, 52	0.14
PT (s) > 13, ≤13	4, 135	2, 58	1.000
PLT (10^9^/L) > 300, ≤300	5, 134	2, 58	1.000
Child-Pugh grade A, B	137,2	59, 1	1.000
MRI features			
Multifocality			0.851
Solitary	115	51	
Multiple	24	9	
L-max > 5, ≤5	53, 86	28, 32	0.333
Tumor margin			1.000
Smooth	31	14	
Non-smooth	108	46	
Tumor-capsule			0.785
Present	124	55	
Absent	15	5	
Peritumoral enhancement			1.000
Present	13	5	
Absent	126	55	
Rim enhancement			0.700
Present	23	12	
Absent	116	48	
Intra-hemorrhage	25, 114	8, 52	0.547
Intra-necrosis	41, 98	22, 38	0.4055
TTPVI			1.000
Present	70	30	
Absent	69	30	
Histologic features			
Histologic grade			0.758
Poorly	31	11	
Moderately	103	46	
Well	5	3	
Satellite nodules			0.693
Present	115	51	
Absent	24	9	
MVI			0.477
Present	132	51	
Absent	17	9	
Early recurrence			0.547
Present	50	25	
Absent	89	35	

ALT = Alanine aminotransferase, AST = Aspartate aminotransferase, ALB = Serum albumin, TBIL = Total bilirubin, DBIL = Direct bilirubin, CR = Creatinine, ALP = Alkaline phosphatase, GGT = γ-glutamyl transpeptadase, PLT = Platelet count, CA199 = Carbohydrate antigen199, CEA = Carcinoembryonic antigen, L-max = Maximum tumor length, TTPVI = Two-trait predictor of venous invasion, MVI = Microvascular invasion.

**Table 3:** Results of univariate and multivariate Cox analyses of the training dataset.

Characteristic	Univariate		Multivariate	
	HR (95%CI)	*p*-Value	HR (95%CI)	*p*-Value
Patient demographics				
Age (y)	1.016 (0.986–1.047)	0.286		
Gender	1.940 (0.698–5.390)	0.204		
Female				
Male				
Liver disease	0.930 (0.525–1.646)	0.803		
Hepatitis B/C virus				
Absent				
Liver cirrhosis	1.275 (0.696–2.335)	0.431		
Present				
Absent				
Ascites	1.904 (0.925–3.919)	0.081		
Present				
Absent				
Surgical treatment	2.139 (1.190–3.843)	0.011	1.047 (0.486–1.876)	0.894
Major hepatectomy				
Minor hepatectomy				
Laboratory factors				
ALB (g/L) > 40, ≤40	0.596 (0.329–1.080)	0.088		
ALT (IU/L) > 50, ≤50	1.440 (0.765–2.711)	0.258		
AST (IU/L) > 40, ≤40	2.324 (1.333–4.050)	0.003	1.975 (1.045–3.732)	0.036
TBIL (μmol/L) > 19, ≤19	1.368 (0.780–2.399)	0.275		
DBIL (μmol/L) > 3.4, ≤3.4	1.688 (0.965–2.952)	0.066		
GGT (IU/L) > 40, ≤40	0.608 (0.348–1.060)	0.079		
ALP (IU/L) > 125, ≤125	1.002 (1.000–1.005)	0.069		
CR (μmol/L) > 110, ≤110	0.994 (0.974–1.014)	0.529		
CEA (ng/mL) > 3.4, ≤3.4	1.209 (0.667–2.190)	0.532		
CA199 (U/mL) > 37, ≤37	2.237 (1.168–4.284)	0.015	1.023 (0.498–2.101)	0.950
PT (s) > 13, ≤13	1.433 (0.993–2.068)	0.055		
PLT (10^9^/L)	0.998 (0.993–1.002)	0.337		
Child-Pugh grade A, B	2.089 (0.288–15.136)	0.466		
MRI features				
Multifocality	2.728 (1.503–4.949)	0.001	1.424 (0.726–2.794)	0.303
Solitary				
Multiple				
L-max > 5, ≤5	1.228 (0.697–2.163)	0.477		
Tumor margin	3.056 (1.213–7.703)	0.018	1.488 (0.563–3.929)	0.422
Smooth				
Non-smooth				
Tumor-capsule	0.205 (0.105–0.398)	<0.001	0.422 (0.198–0.900)	0.026
Present				
Absent				
Peritumoral enhancement	3.215 (1.604–6.448)	0.001	1.183 (0.518–2.704)	0.689
Present				
Absent				
Rim enhancement	6.173(3.438–11.084)	<0.001	2.819 (1.267–6.273)	0.011
Present				
Absent				
Intra-hemorrhage	1.632 (0.853–3.124)	0.139		
Intra-necrosis	1.616 (0.907–2.880)	0.104		
TTPVI	18.061 (6.481–50.333)	<0.001	11.665 (3.978–34.203)	<0.001
Present				
Absent				
Histologic features				
Histologic grade	1.164 (0.756–1.791)	0.491		
Poorly				
Moderately				
Well				
Satellite nodules	1.067 (0.843–1.351)	0.589		
Present				
Absent				
MVI	1.139 (0.844–1.535)	0.395		
Present				
Absent				

ALT = Alanine aminotransferase, AST = Aspartate aminotransferase, ALB = Serum albumin, TBIL = Total bilirubin, DBIL = Direct bilirubin, CR = Creatinine, ALP = Alkaline phosphatase, GGT = γ-glutamyl transpeptadase, PLT = Platelet count, CA199 = Carbohydrate antigen199, CEA = Carcinoembryonic antigen, L-max = Maximum tumor length, TTPVI = Two-trait predictor of venous invasion, MVI = Microvascular invasion.

## Data Availability

Not applicable.

## Abbreviations

AFP-NHCC: AFP-negative (<20 ng/mL) hepatocellular carcinoma; ER: early recurrence; MR: magnetic resonance; RFS: Recurrence-free survival; AUC: area under curve; ROC: receiver operator characteristic; DCA: decision curve analysis.
